# Association between plasma odd-chain fatty acid levels and immune cell traits in psoriasis: insights from a prospective cohort study

**DOI:** 10.3389/fimmu.2025.1500722

**Published:** 2025-04-28

**Authors:** Rongcan Shi, Yifei Xu, Xingyu Jiang, Bo Yu, Rui Ma, Xin Wang, Yuling Shi

**Affiliations:** ^1^ Shanghai Skin Disease Clinical College, Fifth Clinical Medical College, Anhui Medical University, Shanghai, China; ^2^ Department of Dermatology, Shanghai Skin Disease Hospital, Tongji University School of Medicine, Shanghai, China; ^3^ Institute of Psoriasis, Tongji University School of Medicine, Shanghai, China

**Keywords:** psoriasis, odd-chain fatty acid, white blood cell traits, severity, prospective cohort

## Abstract

**Background/Objectives:**

Psoriasis is a chronic, immune-mediated skin disease frequently linked to metabolic dysregulation. Odd-chain fatty acids (OCFAs), a group of bioactive lipids, have been implicated in inflammation and metabolic health; however, their role in psoriasis remains poorly defined. This study aimed to investigate the associations between plasma OCFA levels, white blood cell (WBC) traits, and psoriasis severity.

**Methods:**

A total of 235 patients with moderate-to-severe plaque psoriasis were enrolled from the Shanghai Psoriasis Effectiveness Evaluation CoHort. Baseline plasma OCFA concentrations were measured using gas chromatography–mass spectrometry, and routine hematologic parameters were extracted from clinical records. Psoriasis severity was assessed using the Psoriasis Area and Severity Index, Body Surface Area, Dermatology Life Quality Index, and the Hospital Anxiety and Depression Scale for Anxiety and Depression. Therapeutic response was evaluated at weeks 12 and 28 based on clinical improvement. Multivariate linear and logistic regression analyses, stratified subgroup analyses, and restricted cubic spline models were employed.

**Results:**

Higher plasma levels of C15:0 were significantly associated with increased total WBC and neutrophil counts. C17:0 levels were positively associated with WBC counts among females and older adults, and inversely associated with eosinophil counts in females and individuals with normal BMI. Additionally, C17:1n7 levels were positively associated with lymphocyte and monocyte counts. Total OCFA levels were also positively associated with overall WBC and neutrophil counts. These associations varied by sex, age, BMI, smoking and alcohol consumption history, and the presence of comorbidities such as psoriatic arthritis, hypertension, and type 2 diabetes. While no significant associations were observed between plasma OCFA levels and psoriasis severity or treatment response in the overall cohort, stratified analyses revealed potential relationships in specific subgroups.

**Conclusions:**

Plasma OCFAs are differentially associated with circulating immune cell profiles in patients with psoriasis, suggesting a potential immunomodulatory role. Although OCFAs were not linked to overall disease severity or short-term treatment outcomes, subgroup-specific associations indicate their relevance in particular clinical phenotypes. These findings highlight the need for further longitudinal studies to clarify the role of OCFAs in immune regulation, disease progression, and comorbidity management in psoriasis.

## 1 Introduction

Psoriasis is a chronic, inflammatory, and systemic disease predominantly characterized by erythema and scaling (1). Affecting approximately 125 million people worldwide (2), psoriasis imposes a significant health burden. In China, the number of adult psoriasis patients is estimated to range from 0.9 to 6.1 million, with a central estimate of 2.3 million, placing considerable strain on both individuals and the healthcare system (3). As a multisystem condition, psoriasis is frequently associated with comorbidities such as dyslipidemia, diabetes, and metabolic syndrome (1, 2). Among these, dysregulated lipid metabolism has emerged as a well-established risk factor for disease progression and severity (4–6). Despite significant advances in metabolomics, the relationship between specific fatty acid profiles, psoriasis severity, and therapeutic outcomes remains insufficiently explored.

Fatty acids are broadly categorized into saturated and unsaturated types, and further classified into odd- and even-chain fatty acids (7). While human plasma predominantly contains even-chain fatty acids, odd-chain fatty acids (OCFAs) are present in smaller quantities (7). However, high concentrations of OCFAs, such as pentadecanoic acid (C15:0) and heptadecanoic acid (C17:0), are found in plant oils, fish oils, and ruminant milk fat (7, 8). Emerging evidence suggests that these previously underappreciated fatty acids may play a significant role in the development and progression of various diseases. Specifically, circulating or adipose levels of OCFAs, particularly C15:0 and C17:0, have been linked to cardiovascular disease, diabetes, and metabolic syndrome (9–12). Additionally, C17:1n7, an unsaturated OCFAs not naturally synthesized in mammals, has garnered attention for its potential therapeutic effects.

OCFAs also demonstrate antibiotic, anti-inflammatory, and noncytotoxic immunosuppressive properties (13), which may be particularly relevant to psoriasis. Psoriasis is a chronic inflammatory disorder driven primarily by dysregulated immune responses (1). The anti-inflammatory and immunosuppressive properties of OCFAs suggest that they could modulate immune activity in psoriasis patients, potentially mitigating the excessive inflammation that characterizes the disease. Moreover, psoriasis is often associated with comorbid conditions like metabolic syndrome and dyslipidemia (1, 2), both of which involve abnormalities in lipid metabolism. Given the role of OCFAs in modulating lipid profiles, these fatty acids may influence both the severity and progression of psoriasis. Therefore, exploring the relationship between OCFAs and psoriasis could offer valuable insights into potential therapeutic strategies aimed at modulating immune responses and addressing metabolic disturbances in this chronic inflammatory disease.

In this study, we investigated the relationship between plasma OCFA concentrations, psoriasis severity, and treatment response in patients with moderate-to-severe plaque psoriasis from the prospective cohort study-Shanghai Psoriasis Effectiveness Evaluation CoHort (SPEECH). The study followed patients over a 12- and 28-week period. Recognizing the critical role of immune cells play in the pathogenesis of psoriasis, and previous research highlighting a distinct profile of circulating leukocytes in psoriasis patients (1, 14, 15), we also examined circulating white blood cell (WBC) data from the SPEECH database to explore the potential effects of OCFAs on immune cells profiles in psoriatic patients.

## 2 Methods

### 2.1 Study design

The present analysis utilized both cross-sectional and longitudinal study designs, nested within the Shanghai Psoriasis Effectiveness Evaluation CoHort (SPEECH), as outlined in our previous publications (5, 16). In brief, adult patients diagnosed with chronic moderate-to-severe plaque psoriasis, based on Psoriasis Area and Severity Index (PASI) scores, and considered in need of biologic therapy, were enrolled in SPEECH. SPEECH is an ongoing prospective, multicenter, observational registry designed to investigate the clinical characteristics of psoriasis in the Chinese population and evaluate appropriate diagnostic and treatment strategies for this population (5, 16, 17). Data were collected at seven sites in Shanghai, China, and encompassed various demographic, clinical, and treatment-related data points. To be eligible for SPEECH, patients were required to be aged ≥ 18 years, diagnosed with chronic moderate-to-severe plaque psoriasis, and treated with phototherapy, conventional systemic medications (acitretin or methotrexate), or biologics (adalimumab, ustekinumab, secukinumab, or ixekizumab). Patients who had received conventional systemic treatments or phototherapy within the past 4 weeks, or biologics in the past 12 weeks, were excluded from participation. Additionally, patients with uncontrolled internal medical conditions (e.g., diabetes) or those who were pregnant were also excluded. This prospective cohort study is registered with the Chinese Clinical Trial Registry (ChiCTR2000036186). Informed consent was obtained from all participants, and stringent measures were implemented to protect their privacy and maintain the confidentiality of their data throughout the study.

### 2.2 Participants

**Figure 1:**
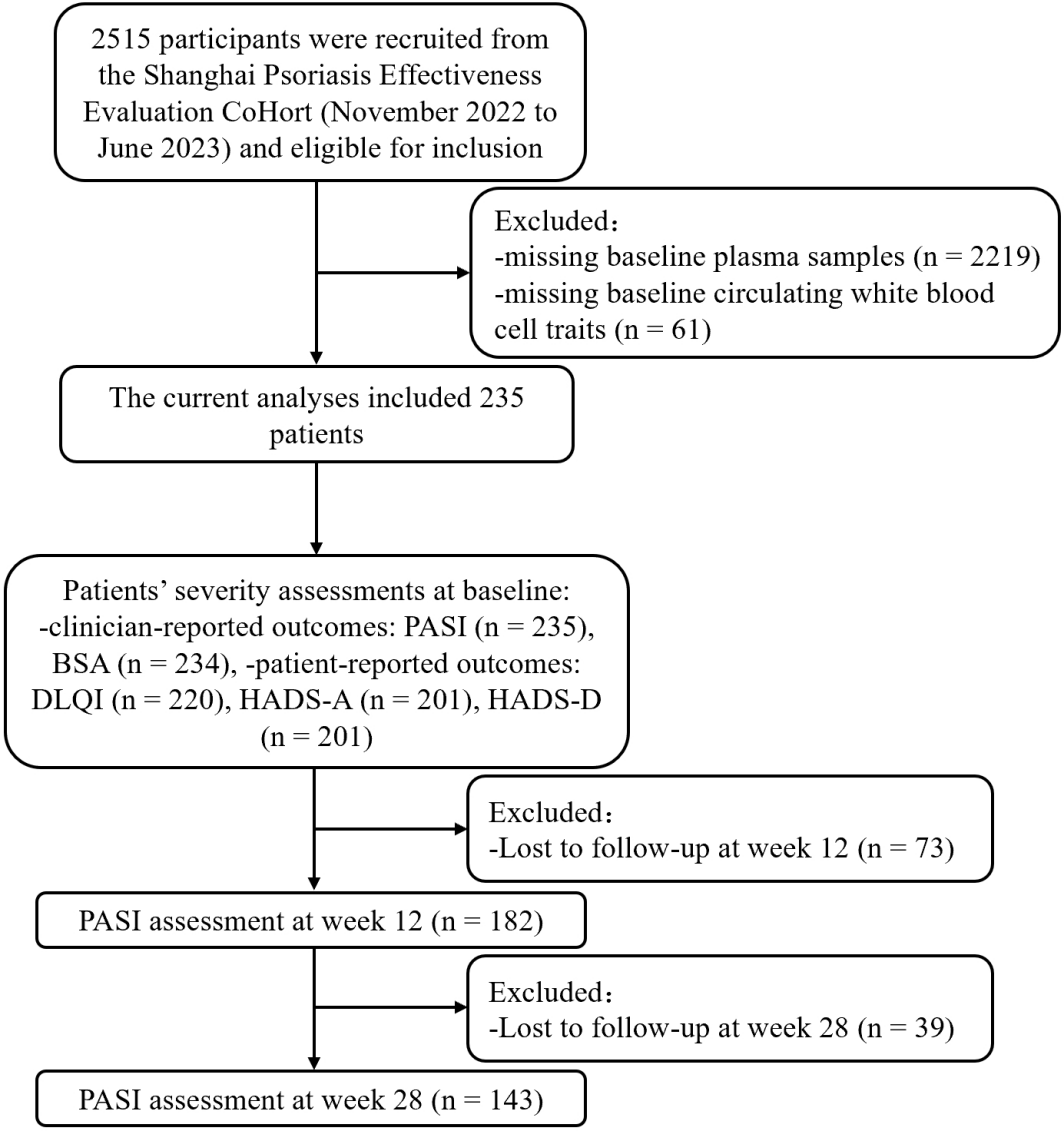
Study flowchart. PASI, Psoriasis Area and Severity Index; BSA, Body Surface Area; DLQI, Dermatology Life Quality Index; HADS-A, hospital anxiety and depression scale for anxiety; HADS-D, hospital anxiety and depression scale for depression.

A total of 2515 patients from the SPEECH study, recruited between November 2022 to June 2023, were initially considered for inclusion in this analysis. Participants were excluded if they met any of the following criteria: 1) missing plasma samples collected at baseline of OCFAs concentration measurement; 2) missing baseline data on circulating white blood cell traits. Consequently, 235 patients were finally included in the study. After enrollment, patients received various treatments, including phototherapy, conventional systemic medications (acitretin or methotrexate), or biological agents (Adalimumab, Ustekinumab, Secukinumab or Ixekizumab). Data, including demographic information and clinical outcomes, were collected at the 12-week and 28-week follow-up visits (**Figure 1**).

### 2.3 Plasma OCFAs detection

The assessment of plasma OCFAs followed a similar procedure to that used for PUFAs, as described in our previous study (5). Briefly, venous peripheral blood plasma was collected from patients at baseline using a standardized protocol. 100 μL plasma was processed for lipid extraction and esterification using established methods (5, 18). Nonadecanoic acid (Sigma, USA) served as the internal standard, while a 37-component fatty acid methyl ester (FAME) mix (37 Component FAME Mix CDAA-252,795, ANPEL Laboratory Technologies (Shanghai) Inc.) was used as the external standard. FAMEs were separated on a capillary column (30 m × 0.25 mm × 0.25 μm) (DB-wax, Agilent Technologies Inc., USA) and analyzed using gas chromatography-mass spectrometry (7890B-5977B, Agilent Technologies Inc., USA). Data were processed using Mass Hunter Software (Agilent Technologies Inc., USA), and the proportions of OCFAs were expressed as molar proportions (mol %) of the total fatty acids. During analysis, plasma samples were organized into batches of up to 22, with two samples from a standard pool included in each batch for quality control (QC). The coefficient of variation for the QC samples were 11.72% for C15:0, 8.14% for C17:0, and 9.88% for C17:1n7, respectively.

### 2.4 Data collection and outcomes

For each patient, demographic information, psoriasis severity and laboratory examinations were collected at baseline, and efficacy outcomes were collected at 12-week follow-up visit. Demographic information included sex, age, body mass index (BMI), education attainment, smoking history, alcohol consumption history, and duration of psoriasis. Psoriasis severity comprised Clinician-Reported Outcomes (ClinROs) and Patient-Reported Outcomes (PROs). Laboratory examinations referred to circulating WBC traits. ClinROs included the PASI, which scores from 0 to a theoretical maximum of 72, with higher scores indicating greater disease severity, and the body surface area (BSA) affected by psoriasis, ranging from 0% to 100% (19). PROs encompassed the Dermatology Life Quality Index (DQLI), which ranges from 0 to 30, with higher scores reflecting greater impairment in quality of life, and Hospital Anxiety and Depression Scale for Anxiety (HADS-A), and Depression (HADS-D) (20, 21). After a 12- and 28-week follow-up, patients were re-evaluated, and the PASI scores were categorized into PASI 50%, PASI 75%, PASI 90%, and PASI 100% to assess the degree of clinical improvement.

Circulating WBC traits were extracted from laboratory examinations conducted at baseline. Specifically, during the baseline period of the study, peripheral venous blood samples were collected from fasting subjects to ensure accurate WBC count measurement. The samples were processed using a Beckman Coulter HMX Hematology Analyzer. Counts of lymphocyte, monocyte, neutrophil, basophil, and eosinophil were measured via a complete blood count, with all values expressed as ×10^3^ cell/μL.

### 2.5 Statistical analysis

Patient characteristics were described using means with standard deviations (SD) for continuous variables, and counts with percentages for categorical variables. The Shapiro-Wilk test was applied to assess the normality of data distribution. Differences between non-normally distributed data were evaluated using the Mann-Whitney U test, while differences in categorical data were assessed using the chi-square test.

To assess the overall effects of OCFAS on outcomes, we calculated the total concentrations of OCFAs by summing the levels of C15:0, C17:0 and C17:1n7. Given the right-skewed distribution of OCFAs levels, we natural log-transformed the concentrations of C15:0, C17:0, C17:1n7, and the total OCFAs when evaluating their associations with psoriasis risk as continuous variables. Additionally, we performed categorical analysis by dividing patients into two groups based on the median values of OCFAs, both individually and in total. Patients were categorized into a low group (< median) and a high group (≥ median). To examine the relationship between plasma OCFAs levels and psoriasis severity as well as circulating WBC traits at baseline, we employed multivariable generalized linear regression models (GLMs). Based on existing the literature, potential confounding factors included sex (male or female), age (years), BMI (normal < 24 kg/m^2^, overweight 24–28 kg/m^2^, obese ≥ 28 kg/m^2^), education attainment (high school or below, college or above), smoking history (yes or no), alcohol consumption history (yes or no), duration of psoriasis (years), and psoriatic comorbidities including psoriatic arthritis, hypertension and type 2 diabetes (5, 17, 22). For the longitudinal study, to determine the relationship between baseline plasma OCFAs levels, PASI scores, and PASI responses at the 12-week follow-up and 28-week follow up, we conducted multivariable logistic regression and multivariable GLMs. In addition to those confounders mentioned above, various treatment, including acitretin, methotrexate, phototherapy, and biologics, were adjusted for in statistical models (5, 16). In this study, we reported β-Coefficients (β) with their corresponding 95% confidence intervals (CIs) for continuous outcomes and odd ratios (ORs) with 95% CI for categorical outcomes, respectively, to illustrate the change in the outcomes.

Several sensitivity analyses were performed to assess the robustness of the findings. First, stratification analyses were conducted based on sex (male, female), age (20–40 years, ≥ 40 years), BMI (< 24, 24-28, ≥ 28 kg/m^2^), smoking history (yes, no), and alcohol consumption history (yes, no). Second, restricted cubic spline (RCS) analyses were used to explore potential nonlinear dose-response relationships between OCFAs and various outcomes. Third, sensitivity analyses were carried out by excluding participants with psoriatic arthritis, hypertension, or type 2 diabetes to examine the impact of these comorbidities on the results. Lastly, multiple imputation was applied to handle missing covariate data. Specifically, the missing values were as follows: 1 (0.43%) for BMI, 7 (2.98%) for education attainment, 23 (9.79%) for the duration of psoriasis, 1 (0.43%) for psoriatic arthritis, 3 (1.28%) for hypertension, 3 (1.28%) for type 2 diabetes.

All analyses were performed using SAS 9.4 software (SAS Institute Inc., Cary, NC, USA) and R (version 4.3.1, R Development Core Team). RCS analyses were conducted using the R packages “rms”. The significance level was set at a two-sided *P*-value < 0.05.

## 3 Results

### 3.1 Patients baseline characteristics

The baseline characteristics of the study participants are summarized in **Table 1**. Among the 235 participants, 182 (77.4%) were male, and 53 (22.6%) were female, with a mean age 51.57 ± 15.88 years. As shown in **Supplementary Table S1**, the median (Q1, Q3) concentrations of C15:0, C17:0, C17:1n7, and total OCFAs were 0.24 (0.19, 0.30), 0.46 (0.42, 0.52), 0.20 (0.16, 0.25), and 0.90 (0.79, 1.08) mol%, respectively.

**Table 1:** Baseline characteristics of the study population by medians of odd-chain fatty acids (mol %).

Characteristic	Total	C15:0			C17:0			C17:1n7			Total OCFA		
		Low (<0.24)	High (≥0.24)	*P*-value	Low (<0.46)	High (≥0.46)	*P*-value	Low (<0.20)	High (≥0.20)	*P*-value	Low (<0.90)	High (≥0.90)	*P*-value
N	235	117	118		117	118		118	117		117	118	
Age (year)				0.853			0.918			0.918			0.918
<40	67 (28.5)	34 (29.1)	33 (28.0)		33 (28.2)	34 (28.8)		34 (28.8)	33 (28.2)		33 (28.2)	34 (28.8)	
≥40	168 (71.5)	83 (70.9)	85 (72.0)		84 (71.8)	84 (71.2)		84 (71.2)	84 (71.8)		84 (71.8)	84 (71.2)	
Sex (male)	182 (77.4)	94 (80.3)	88 (74.6)	0.290	93 (79.5)	89 (75.4)	0.456	89 (75.4)	93 (79.5)	0.456	92 (78.6)	90 (76.3)	0.665
Education attainment				0.047			0.166			0.283			0.135
High school or lower	132 (57.9)	74 (64.4)	58 (51.3)		70 (62.5)	62 (53.4)		70 (61.4)	62 (54.4)		71 (62.8)	61 (53.0)	
College or above	96 (42.1)	41 (35.6)	55 (48.7)		42 (37.5)	54 (46.6)		44 (38.6)	52 (45.6)		42 (37.2)	54 (47.0)	
BMI (kg/m^2^)				0.638			0.090			0.041			0.632
<24	93 (39.7)	50 (42.7)	43 (36.7)		43 (36.7)	50 (42.7)		56 (47.5)	37 (31.9)		50 (42.7)	43 (36.8)	
24-28	98 (41.9)	47 (40.2)	51 (44.6)		46 (39.3)	52 (44.4)		45 (38.1)	53 (45.7)		46 (39.3)	52 (44.4)	
≥28	43 (18.4)	20 (17.1)	23 (19.7)		28 (24.0)	15 (12.8)		17 (14.4)	26 (22.4)		21 (18.0)	22 (18.8)	
Smoking history (yes)	139 (59.2)	74 (63.3)	65 (55.1)	0.203	72 (61.5)	67 (56.8)	0.458	65 (55.1)	74 (63.3)	0.203	71 (60.7)	68 (57.6)	0.634
Alcohol consumption history (yes)	81 (34.5)	45 (38.5)	36 (30.5)	0.200	42 (35.9)	39 (33.1)	0.646	40 (33.9)	41 (35.0)	0.854	42 (35.9)	39 (33.1)	0.646
Psoriasis duration (year)	15.9 ± 12.9	15.3 ± 14.0	16.6 ± 11.7	0.133	14.8 ± 10.6	17.0 ± 14.8	0.620	15.6 ± 13.2	16.3 ± 12.5	0.592	14.1 ± 10.6	17.8 ± 14.7	0.125
Comorbidities													
Psoriatic arthritis (yes)	30 (12.88)	15 (12.82)	15 (12.93)	0.980	15 (12.82)	15 (12.93)	0.980	15 (12.71)	15 (13.04)	0.940	15 (12.82)	15 (12.93)	0.980
Hypertension (yes)	77 (33.62)	43 (37.39)	34 (29.83)	0.060	41 (35.34)	36 (31.85)	0.852	35 (30.44)	42 (36.84)	0.478	40 (35.08)	37 (32.18)	0.881
Type 2 diabetes(yes)	34 (14.72)	26 (22.41)	8 (6.96)	0.004	19 (16.38)	15 (13.04)	0.258	18 (15.52)	16 (13.91)	0.931	20 (17.39)	14 (12.06)	0.477

Data are shown as n (%) or mean ± SD.

*P*-values are based on any differences between groups examined using the Mann-Whitney U test for non-normally distributed continuous variables, chi-squared test for categorical data.

OCFA, odd-chain fatty acid; SD, standard deviation.

Participants were stratified into low and high group based on the median values of C15:0, C17:0, C17:1n7, and total OCFAs levels. Notably, individuals with lower C15:0 levels were more likely to have higher education levels (*P* < 0.05) and a higher incidence of type 2 diabetes (*P* < 0.05). Additionally, participants with higher C17:1n7 levels were more likely to be obese (*P* < 0.05). No other significant differences in demographic or clinical characteristics were observed between the groups (all *P* > 0.05).

### 3.2 Associations between plasma OCFAs levels, circulating WBC traits, and psoriasis severity at baseline

**Figure 2:**
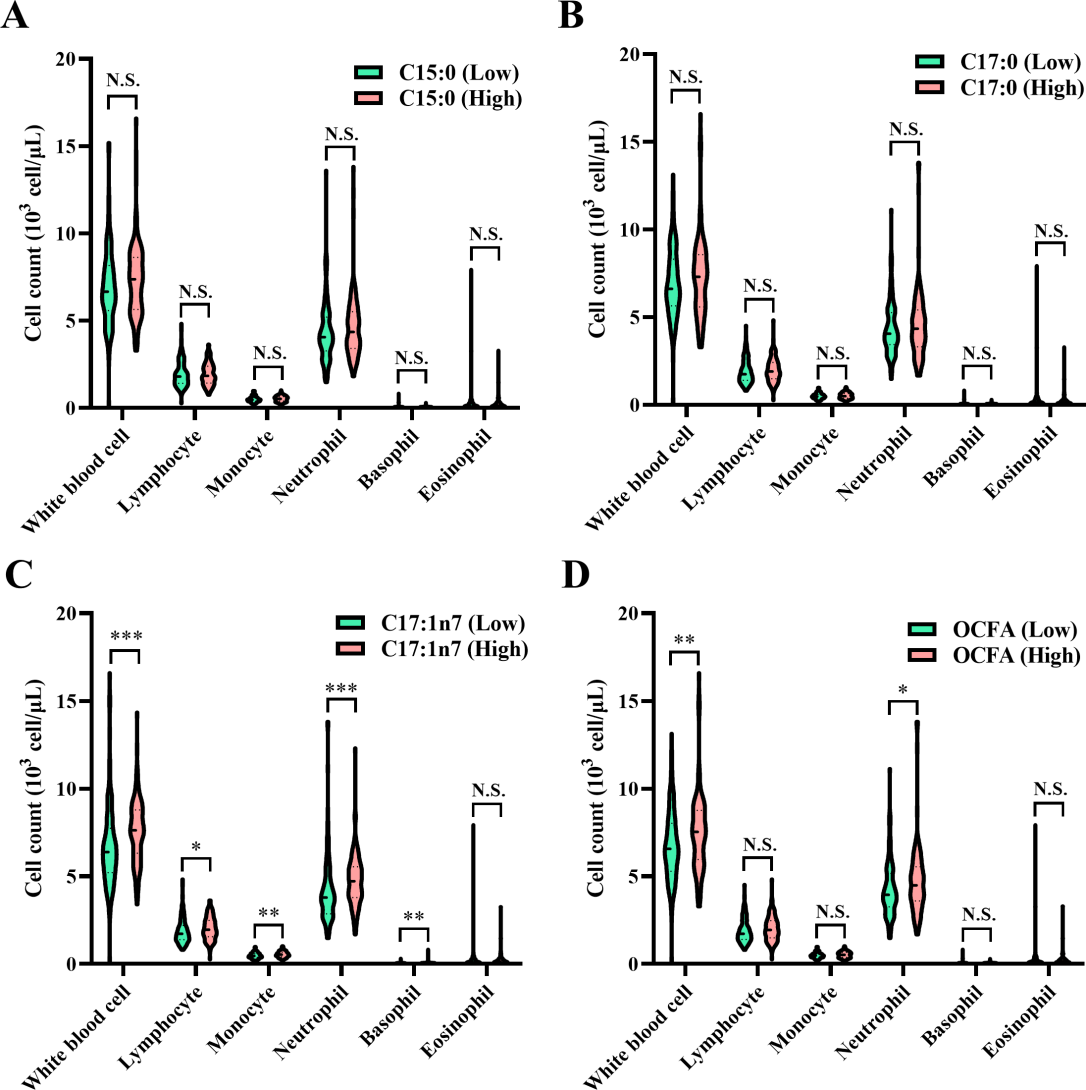
Comparison of circulating white blood cell traits among patients categorized into low and high groups based on the median values of OCFAS. **(A)** C15:0; **(B)** C17:0; **(C)** C17:1n7; **(D)** total OCFAs. Association between plasma odd-chain FAs levels and white blood cell counts in normal or psoriasis participants. Statistical comparisons between groups, ^*^ *P* < 0.05, ^**^ *P* < 0.01, ***P < 0.001, N.S., no significant difference. OCFA, odd-chain fatty acid.

As illustrated in **Figure 2**, patients with elevated C17:0 levels exhibited significantly lower WBC, lymphocyte, and neutrophil counts (all *P* < 0.05), while showing higher monocyte and basophil counts (all *P* < 0.05). Conversely, patients with higher total OCFAs levels had significantly increased WBC and neutrophil counts (all *P* < 0.05).

As shown in **Table 2**, C15:0 levels were positively associated with WBC counts (β = 1.28, 95% CI: 0.44–2.13, *P* = 0.003) and neutrophil counts (β = 0.82, 95% CI: 0.12–1.51, *P* = 0.021). Additionally, C17:1n7 levels were positively associated with lymphocyte counts (β = 0.35, 95% CI: 0.11–0.59, *P* = 0.005) and monocyte counts (β = 0.07, 95% CI: 0.01–0.14, *P* = 0.021). A positive association was also observed between total OCFAs levels and total WBC counts (β = 1.51, 95% CI: 0.28–2.73, *P* = 0.016).

**Table 2:** Association between plasma OCFAs levels and circulating WBC traits in psoriasis participants (n = 235).

Categories	WBC	Lymphocyte	Monocyte	Neutrophil	Basophil	Eosinophil
C15:0 levels						
Low	Ref.	Ref.	Ref.	Ref.	Ref.	Ref.
High	0.50 (-0.07, 1.07)	-0.00 (-0.18, 0.18)	0.03 (-0.02, 0.07)	0.39 (-0.07, 0.86)	-0.00 (-0.02, 0.02)	-0.03 (-0.20, 0.13)
Continuous (ln C15:0)	1.28 (0.44, 2.13)^**^	0.16 (-0.11, 0.44)	0.04 (-0.03, 0.11)	0.82 (0.12, 1.51)^*^	-0.01 (-0.03, 0.02)	-0.23 (-0.47, 0.01)
C17:0 levels						
Low	Ref.	Ref.	Ref.	Ref.	Ref.	Ref.
High	0.49 (-0.07, 1.05)	0.10 (-0.08, 0.27)	0.02 (-0.02, 0.07)	0.27 (-0.19, 0.72)	-0.00 (-0.02, 0.01)	-0.07 (-0.23, 0.09)
Continuous (ln C17:0)	1.15 (-0.47, 2.77)	0.29 (-0.23, 0.80)	0.02 (-0.12, 0.15)	0.57 (-0.75, 1.89)	-0.01 (-0.06, 0.03)	-0.22 (-0.67, 0.23)
C17:1n7 levels						
Low	Ref.	Ref.	Ref.	Ref.	Ref.	Ref.
High	0.75 (0.21, 1.30)^**^	0.15 (-0.03, 0.32)	0.05 (0.01, 0.10)^*^	0.43 (-0.02, 0.88)	0.01 (-0.01, 0.02)	-0.06 (-0.22, 0.10)
Continuous (ln C17:1n7)	0.78 (-0.00, 1.55)	0.35 (0.11, 0.59)^**^	0.07 (0.01, 0.14)^*^	0.31 (-0.33, 0.94)	0.01 (-0.01, 0.03)	-0.01 (-0.23, 0.21)
OCFAs levels						
Low	Ref.	Ref.	Ref.	Ref.	Ref.	Ref.
High	0.76 (0.21, 1.31)^**^	0.13 (-0.05, 0.30)	0.03 (-0.02, 0.07)	0.48 (0.03, 0.93)^*^	-0.00 (-0.02, 0.01)	-0.05 (-0.20, 0.11)
Continuous (ln OCFAs)	1.51 (0.28, 2.73)^*^	0.37 (-0.02, 0.76)	0.07 (-0.03, 0.17)	0.83 (-0.17, 1.83)	-0.00 (-0.04, 0.03)	-0.19 (-0.54, 0.16)

Data expressed as β (95% confidence interval). Significant results were presented in bold. The regression coefficients were adjusted for age, sex, BMI, education attainment, smoking history, alcohol consumption history, psoriasis duration, and psoriatic comorbidities including psoriatic arthritis, hypertension and type 2 diabetes. ^*^ *P* < 0.05, ^**^ *P* < 0.01. BMI, body mass index; ln, natural log-transformed; OCFA, odd-chain fatty acid; WBC, white blood cell.

In categorical analysis, patients in the high C17:1n7 group had significantly higher WBC (high vs. low, β = 0.75, 95% CI: 0.20–1.31, *P* = 0.008) and monocyte counts (high vs. low, β = 0.05, 95% CI: 0.01–0.10, *P* = 0.022) compared to the low group. Similarly, higher neutrophil counts were observed in the high total OCFAs group compared to the low group (high vs. low, β = 0.48, 95% CI: 0.03–0.93, *P* = 0.036).

**Figure 3:**
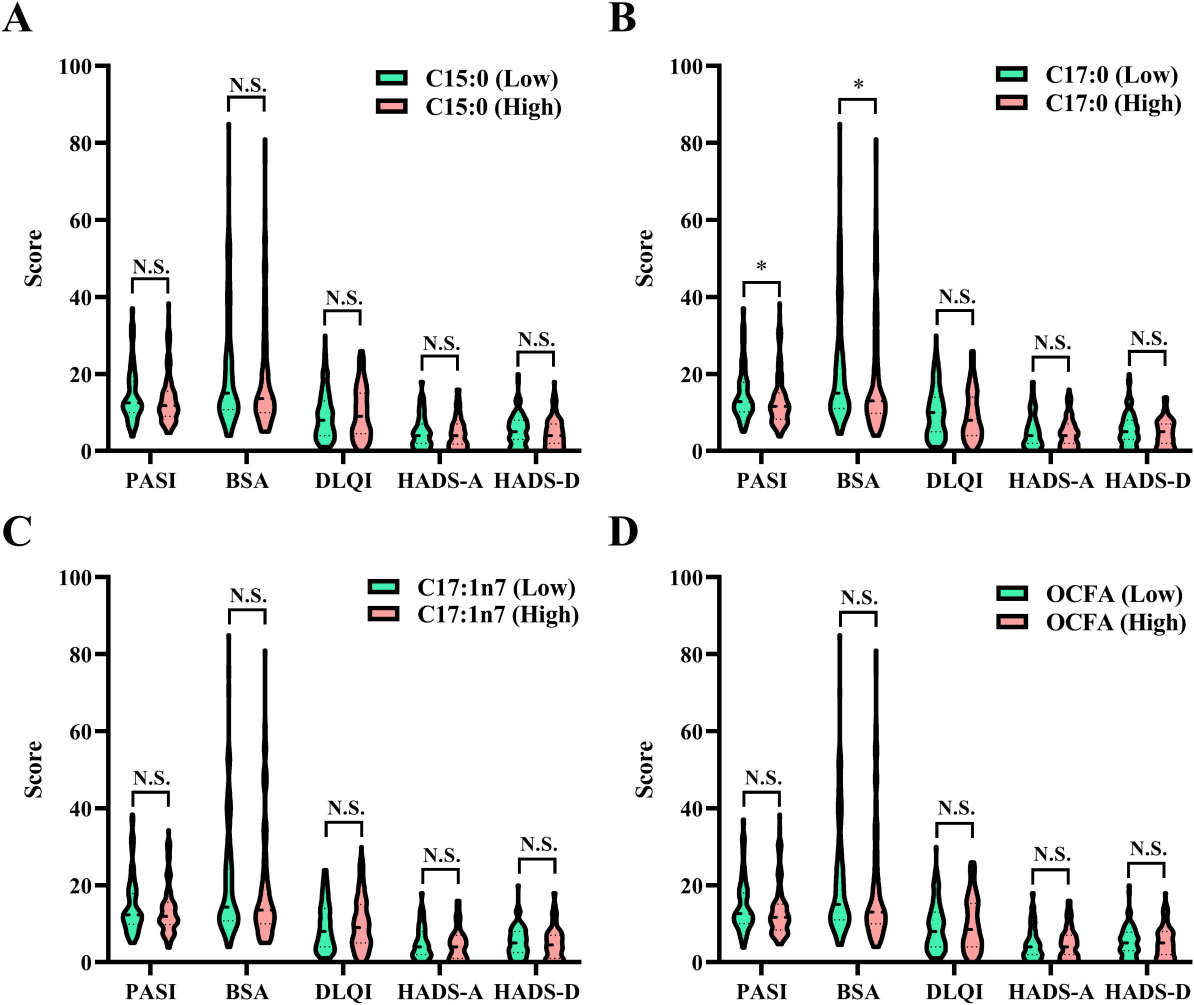
Comparison of psoriasis severity among patients categorized into low and high groups based on the median values of OCFAS. **(A)** C15:0; **(B)** C17:0; **(C)** C17:1n7; **(D)** total OCFAs. Statistical comparisons between groups, ^*^ *P* < 0.05, N.S., no significant difference. BSA, body surface area; DLQI, dermatology life quality index; HADS-A, hospital anxiety and depression scale for anxiety; HADS-D, hospital anxiety and depression scale for depression; OCFA, odd-chain fatty acid; PASI, psoriasis area and severity index.

As shown in **Figure 3**, patients with higher C17:0 levels had significantly lower PASI and BSA scores (all *P* < 0.05), suggesting a potential association with reduced psoriasis severity. However, as presented in **Table 3**, no significant associations were found between OCFAs levels and psoriasis severity in the overall cohort. Despite clear relationships with WBC traits, OCFAs levels did not show a statistically significant impact on psoriasis severity cross all participants.

**Table 3:** Association between plasma OCFAs levels and psoriasis severity in psoriasis participants (n = 235).

Categories	PASI	BSA	DLQI	HADS-A	HADS-D
C15:0 levels					
Low	Ref.	Ref.	Ref.	Ref.	Ref.
High	-0.88 (-2.63, 0.87)	-1.78 (-5.73, 2.17)	0.26 (-1.50, 2.02)	-0.44 (-1.67, 0.79)	-0.69 (-1.86, 0.48)
Continuous (ln C15:0)	-1.52 (-4.15, 1.11)	-1.08 (-7.02, 4.86)	1.00 (-1.58, 3.59)	-0.25 (-2.06, 1.56)	-1.12 (-2.84, 0.60)
C17:0 levels					
Low	Ref.	Ref.	Ref.	Ref.	Ref.
High	-1.15 (-2.86, 0.56)	-2.33 (-6.19, 1.53)	-1.01 (-2.72, 0.70)	-0.94 (-2.14, 0.25)	-1.00 (-2.15, 0.14)
Continuous (ln C17:0)	-2.68 (-7.65, 2.28)	-7.97 (-19.31, 3.36)	-2.35 (-7.22, 2.52)	-1.80 (-5.19, 1.60)	-2.48 (-5.72, 0.75)
C17:1n7 levels					
Low	Ref.	Ref.	Ref.	Ref.	Ref.
High	-1.44 (-3.15, 0.26)	-2.59 (-6.43, 1.26)	0.64 (-1.07, 2.34)	-0.37 (-1.57, 0.83)	-0.38 (-1.52, 0.77)
Continuous (ln C17:1n7)	-1.24 (-3.63, 1.15)	-2.11 (-7.55, 3.32)	1.31 (-1.04, 3.66)	-0.33 (-1.98, 1.32)	-0.62 (-2.19, 0.95)
OCFAs levels					
Low	Ref.	Ref.	Ref.	Ref.	Ref.
High	-1.22 (-2.92, 0.49)	-1.71 (-5.56, 2.13)	0.16 (-1.54, 1.86)	0.03 (-1.17, 1.23)	-0.18 (-1.33, 0.96)
Continuous (ln OCFAs)	-2.45 (-6.24, 1.34)	-4.46 (-13.08, 4.16)	0.43 (-3.30, 4.16)	-0.90 (-3.50, 1.69)	-1.72 (-4.19, 0.76)

Data expressed as β (95% confidence interval). The regression coefficients were adjusted for age, sex, BMI, education attainment, smoking history, alcohol consumption history, psoriasis duration, and psoriatic comorbidities including psoriatic arthritis, hypertension and type 2 diabetes. BMI, body mass index; BSA, body surface area; DLQI, dermatology life quality index; HADS-A, hospital anxiety and depression scale for anxiety; HADS-D, hospital anxiety and depression scale for depression; ln, natural log-transformed; OCFA, odd-chain fatty acid; PASI, psoriasis area and severity index.

Additionally, as shown in **Supplementary Figure S1**, a significant nonlinear association was observed between C15:0 levels and eosinophil counts (*P*_overall_ = 0.001, *P*_nonlinear_ = 0.003).

### 3.3 Stratified analysis of plasma OCFAs levels, circulating WBC traits, and psoriasis severity at baseline

To explore the potential modifying effects of demographic and clinical factors, we conducted stratification analyses assessing the associations between plasma OCFA levels, circulating WBC traits, and psoriasis severity at baseline. The stratification factors included sex, age, BMI, smoking history, alcohol consumption history, psoriatic arthritis, hypertension and type 2 diabetes.

As presented in **Supplementary Table S2**, plasma C15:0 levels were positively associated with total WBC counts in females, individuals over 40 years old, patients with a BMI of < 24 kg/m^2^ or 24–28 kg/m^2^, those with a smoking history, or those without a history of alcohol consumption, psoriatic arthritis, or type 2 diabetes (β = 2.59, 95% CI: 0.87–4.30; β = 1.36, 95% CI: 0.28–2.43; β = 1.60, 95% CI: 0.18–3.01; β = 1.28, 95% CI: 0.17–2.38; β = 1.43, 95% CI: 0.22–2.64; β = 1.40, 95% CI: 0.21–2.59; β = 1.47, 95% CI: 0.60–2.34; β = 1.00, 95% CI: 0.09–1.92, respectively). In younger participants (< 40 years), higher C15:0 levels were positively associated with lymphocyte counts (β = 0.58, 95% CI: 0.12–1.04). Additionally, individuals aged > 40 years, those with a BMI of 24–28 kg/m^2^, or those without psoriatic arthritis exhibited increased neutrophil counts in association with elevated C15:0 levels (β = 1.02, 95% CI: 0.13–1.90; β = 1.01, 95% CI: 0.12–1.90; β = 0.86, 95% CI: 0.16–1.56). Conversely, negative associations were observed between C15:0 and eosinophil counts in females, older adults, individuals with a BMI < 24 kg/m^2^, smokers, or those without a history of alcohol consumption (β = -1.10, 95% CI: -2.05–0.15; β = -0.33, 95% CI: -0.63–0.02; β = -0.56, 95% CI: -1.02–0.10; β = -0.48, 95% CI: -0.85–0.11; β = -0.52, 95% CI: -0.89–0.15, respectively).

For C17:0, positive relationships with total WBC counts were found in females and older individuals (β = 5.06, 95% CI: 0.33–9.79; β = 2.44, 95% CI: 0.13–4.75), whereas a negative association was noted between C17:0 and eosinophil counts in females (β = -2.87, 95% CI: -5.31–0.43) (**Supplementary Table S3**).

C17:1n7 was positively associated with total WBC counts in females, individuals with an overweight BMI (24–28 kg/m^2^), those without a history of alcohol consumption, and those without psoriatic arthritis (β = 2.06, 95% CI: 0.04–4.08; β = 1.19, 95% CI: 0.24–2.13; β = 1.12, 95% CI: 0.04–2.20; β = 0.85, 95% CI: 0.04–1.67). Additionally, C17:1n7 levels were positively associated with lymphocyte counts in non-smokers, non-drinkers, or individuals without psoriatic arthritis or Type 2 diabetes (β = 0.49, 95% CI: 0.14–0.85; β = 0.45, 95% CI: 0.14–0.77; β = 0.42, 95% CI: 0.16–0.67; β = 0.49, 95% CI: 0.17–0.80; β = 0.33, 95% CI: 0.06–0.59). Positive associations were also found between C17:1n7 and monocyte counts in males, older adults, smokers, or individuals without psoriatic arthritis (β = 0.08, 95% CI: 0.00–0.15; β = 0.08, 95% CI: 0.00–0.16; β = 0.11, 95% CI: 0.00–0.21; β = 0.07, 95% CI: 0.00–0.14) (**Supplementary Table S4**).

Total OCFAs levels exhibited significant positive associations with total WBC counts in female, older adults, individuals without a history of alcohol consumption, or those without psoriatic arthritis (β = 4.15, 95% CI: 1.21–7.09; β = 2.11, 95% CI: 0.42–3.80; β = 1.89; 95% CI: 0.12–3.66; β = 1.78, 95% CI: 0.50–3.05). Moreover, total OCFAs levels were positively associated with neutrophil counts in older adults (β = 1.47, 95% CI: 0.07–2.87) and lymphocyte counts in individuals without psoriatic arthritis (β = 0.50, 95% CI: 0.09–0.91), whereas a negative association was observed with eosinophil counts in females (β = -1.66, 95% CI: -3.28–0.05) (**Supplementary Table S5**).

Regarding psoriasis severity, the associations were more limited. Negative relationships were observed between C15:0 levels and HADS-D scores in individuals with an overweight BMI (24–28 kg/m^2^) (β = -3.37, 95% CI: -6.61–0.13). Similarly, C17:0 levels were negatively associated with PASI scores in females (β = -10.22, 95% CI: -20.27–0.18) and with HADS-A scores in younger adults (β = -5.33, 95% CI: -10.15–0.51) (**Supplementary Tables S6**-**S9**).

Overall, these results highlight specific associations between OCFA levels and circulating WBC traits, which vary across demographic and clinical subgroups. Additionally, while associations with psoriasis severity were more limited, some evidence suggests a potential role of OCFA levels in modulating psoriasis-related psychological and inflammatory parameters.

### 3.4 Associations between plasma OCFAs levels and PASI scores/treatment response at 12-week and 28-week follow-up

To assess the potential association between plasma OCFA levels and treatment response in psoriatic patients, we performed multivariable logistic regression models and multivariable generalized linear regression analyses. These models investigated the relationships between C15:0, C17:0, C17:1n7, and total OCFAs with PASI50, PASI75, PASI90, PASI100, and PASI scores at 12-week and 28-week follow-up visit.

As shown in **Table 4** and **Table 5**, no statistically significant associations were observed between plasma OCFAs levels and PASI scores or treatment response at either time point.

**Table 4:** Associations between plasma OCFAs levels and PASI scores/response at 12-week follow-up visit (n = 182).

Categories	PASI 50	PASI 75	PASI 90	PASI 100	PASI
	OR (95% CI)	OR (95% CI)	OR (95% CI)	OR (95% CI)	β (95% CI)
C15:0	2.43 (0.64, 9.16)	1.59 (0.54, 4.65)	1.19 (0.43, 3.30)	1.58 (0.48, 5.17)	-0.61 (-2.92, 1.69)
C17:0	1.89 (0.18, 19.75)	1.07 (0.15, 7.83)	0.90 (0.12, 6.64)	0.22 (0.02, 2.44)	-3.59 (-7.92, 0.74)
C17:1n7	1.52 (0.47, 4.92)	0.69 (0.27, 1.75)	0.85 (0.34, 2.11)	1.44 (0.47, 4.42)	-1.29 (-3.33, 0.75)
OCFAs	2.41 (0.38, 15.41)	1.05 (0.23, 4.76)	0.96 (0.22, 4.18)	1.01 (0.17, 6.09)	-2.25 (-5.52, 1.02)

The multivariable logistic regression models and multivariable generalized linear models were adjusted for age, sex, BMI, education attainment, smoking history, alcohol consumption history, psoriasis duration, psoriatic comorbidities including psoriatic arthritis, hypertension and type 2 diabetes, and treatment. CI, confidence interval; OR, odd ratio; PASI, psoriasis area and severity index.

**Table 5:** Associations between plasma OCFAs levels and PASI scores/response at 28-week follow-up visit (n = 143).

Categories	PASI 50	PASI 75	PASI 90	PASI 100	PASI
	OR (95% CI)	OR (95% CI)	OR (95% CI)	OR (95% CI)	β (95% CI)
C15:0	1.27 (0.30, 5.30)	1.09 (0.32, 3.75)	1.19 (0.38, 3.71)	1.35 (0.38, 4.76)	0.23 (-1.65, 2.11)
C17:0	0.24 (0.02, 3.84)	0.34 (0.03, 3.40)	0.47 (0.05, 4.15)	0.33 (0.02, 4.69)	-1.29 (-4.92, 2.33)
C17:1n7	0.47 (0.12, 1.88)	0.45 (0.14, 1.46)	0.81 (0.27, 2.40)	0.68 (0.18, 2.57)	-1.18 (-2.84, 0.49)
OCFAs	0.48 (0.06, 3.71)	0.49 (0.08, 2.86)	0.81 (0.16, 4.16)	0.76 (0.11, 5.52)	-0.96 (-3.65, 1.72)

The multivariable logistic regression models and multivariable generalized linear models were adjusted for age, sex, BMI, education attainment, smoking history, alcohol consumption history, psoriasis duration, psoriatic comorbidities including psoriatic arthritis, hypertension and type 2 diabetes, and treatment. CI, confidence interval; OR, odd ratio; PASI, psoriasis area and severity index.

## 4 Discussion

Our study provides novel insights into the associations between plasma levels of OCFA and both circulating WBC traits and psoriasis severity. We identified several significant associations between specific OCFAs and distinct WBC subtypes, with these relationships varying by demographic and clinical characteristics. However, despite these clear immunological associations, we did not observe a strong association between OCFAs and overall psoriasis severity, suggesting a more nuanced role of OCFAs in disease modulation.

OCFAs such as C15:0 and C17:0, predominantly derived from dietary sources like dairy, fish, and certain plant oils, have been reported to exert anti-inflammatory effects in a range of conditions, including inflammatory bowel disease, metabolic syndrome, and type 2 diabetes (23–25). Nonetheless, limited research has explored their influence on immune cell populations. Our findings reveal differential associations between individual OCFAs and WBC subtypes, implying a potential immunomodulatory role. Specially, elevated plasma levels of C15:0 were positively associated with WBC and neutrophil counts, while C17:1n7 was correlated with increased lymphocyte and monocyte counts. These results support the hypothesis that OCFAs may influence immune cell composition, possibly by modulating inflammatory signaling pathways.

The immune system in psoriasis is characterized by dysregulated WBC profiles, particularly increased neutrophils, which are frequently elevated during active disease phases (26). The observed positive association between C15:0 and neutrophil counts aligns with prior studies linking neutrophil infiltration to psoriasis pathogenesis (27). Neutrophils contribute to disease progression by releasing pro-inflammatory cytokines, including TNF-α, IL-17, and IL-23—key mediators in psoriasis (27). Similarly, monocytes, which can differentiate into macrophages, are also integral to psoriasis-related inflammation (28). The positive relationship between C17:1n7 and monocyte counts may reflect its involvement in monocytes/macrophage differentiation or activation, although further mechanistic studies are needed. Interestingly, C17:0 was associated with reduced WBC counts in several subgroups, suggesting a potential anti-inflammatory effect that may be compartmentalized within certain immune cell populations. This aligns with prior studies indicating that anti-inflammatory properties of OCFAs, although the precise molecular mechanisms remain largely undefined.

Notably, while C15:0 has been recognized for its anti-inflammatory effects in other disease contexts—ameliorating inflammation, anemia, dyslipidemia, inflammatory bowel disease, and fibrosis *in vivo* (23, 29)—our findings suggest a potentially pro-inflammatory role in psoriasis, as indicated by its association with elevated neutrophil counts. This apparent discrepancy could stem from the complex and context-dependent roles of fatty acids in inflammation, where the immunological effects of C15:0 may differ based on disease type, tissue microenvironment, or systemic inflammatory status. Conversely, the role of C17:0 in immune modulation has been minimally studied. To our knowledge, this is the first report indicating that C17:0 may exert anti-inflammatory effects in psoriasis, as evidenced by its inverse association with total WBC counts in certain subgroups. These findings warrant further exploration into C17:0 as a potential immunomodulatory biomarker.

Despite the robust associations between OCFAs and WBC traits, their relationship with psoriasis severity, as measured by PASI scores, was less evident. We did not find significant correlations between overall plasma OCFA levels and disease severity. However, subgroup analyses revealed intriguing patterns: in overweight individuals, higher C15:0 levels were inversely associated with depression scores, suggesting a potential link between OCFAs and psychological well-being. Similarly, C17:0 was inversely associated with PASI scores in females and with anxiety scores in younger adults, indicating that certain OCFAs may influence psychological stress responses or inflammation-related disease exacerbation. These findings support the notion that OCFAs may affect psoriasis outcomes indirectly—through both immune regulation and modulation of psychological stress, which is a recognized exacerbating factor in psoriasis. While immune cells such as T cells are central to psoriasis pathogenesis, most prior OCFA-related studies have focused on their associations with disease risk rather than disease severity (30). This suggests that future research should explore the potential role of OCFAs in psoriasis onset and progression, which may offer opportunities for preventive strategies or early intervention.

The underlying mechanisms linking OCFAs to immune traits and psoriasis severity are likely multifactorial, involving immune signaling cascades and metabolic regulation. OCFAs such as C15:0 have been shown to influence immune function and cytokine expression (31), potentially via modulation of key inflammatory pathways, including NF-κB and MAPK (32, 33). These pathways regulate immune cell differentiation and activation—processes fundamental to psoriasis (34). Additionally, OCFAs may interact with the STAT3 pathway, which plays a critical role in psoriasis pathogenesis (35). A previous study reported that C15:0 could suppress IL-6-induced JAK2/STAT3 signaling in MCF-7/stem-like breast cancer cells (36). This raises the possibility that OCFAs may exert context-specific effects on psoriasis by modulating STAT3 or other cytokine signaling pathways. Moreover, their influence on lipid signaling and membrane dynamics could also alter immune cell function (37). It is also important to consider the impact of comorbid conditions—such as obesity, diabetes, and psoriatic arthritis—and genetic predispositions, which may modify the relationship between OCFAs and disease severity. The variation in associations across demographic subgroups underscores the complexity of these interactions.

This study has several notable strengths. First, it utilizes data from a well-characterized prospective psoriasis cohort, enabling the assessment of both cross-sectional and longitudinal relationships. Second, the use of high-resolution mass spectrometry provides reliable quantification of plasma OCFA levels. Third, while prior research has predominantly focused on common fatty acids—such as even-chain saturated, monounsaturated, or polyunsaturated fatty acids (5, 38, 39)—our study is the first to investigate the associations between OCFAs, WBC traits, and psoriasis severity. Additionally, stratified and sensitivity analyses based on sex, age, BMI, smoking and alcohol history, and comorbidities (psoriatic arthritis, hypertension, and diabetes) offer valuable insight into effect modification by demographic and clinical factors.

However, certain limitations should be acknowledged. The relatively small sample size, particularly after subgroup stratification, may reduce statistical power. The follow-up duration of 12 or 28 weeks may also be insufficient to fully capture the long-term effects of OCFAs on disease severity or treatment response. Furthermore, the absence of follow-up measurements for OCFA levels and immune traits limits our ability to assess dynamic changes over time. Additionally, the potential impact of unmeasured or missing covariates, such as detailed genetic factors, or specific lifestyle behaviors (e.g., diet or exercise), may have influenced the observed associations and should be considered when interpreting the findings.

In conclusion, our findings suggest that OCFAs are associated with distinct immune cell traits in individuals with psoriasis and may play a role in immune modulation. While their influence on overall disease severity remains unclear, associations in specific subgroups suggest that OCFAs could indirectly impact psoriasis outcomes through effects on immune function and psychological well-being. Future studies, including mechanistic and interventional research, are needed to validate these observations and evaluate the therapeutic potential of OCFAs in psoriasis management.

## Data Availability

The original contributions presented in the study are included in the article/**Supplementary Material**. Further inquiries can be directed to the corresponding author.
