# Supplementary material for: Association between plasma odd-chain fatty acid levels and immune cell traits in psoriasis: insights from a prospective cohort study
Source: Front Immunol. 2025 Apr 28;16:1500722. doi: 10.3389/fimmu.2025.1500722 (PMC12066453; doi:10.3389/fimmu.2025.1500722)
Supplement: Supplementary file 1 [file DataSheet1.docx]

Supplementary Material

# Supplementary Figures and Tables

## Supplementary Tables

Table S1. Baseline plasma odd-chain fatty acid concentrations (mol%) in patients with psoriasis (n = 235)

| **OCFAs** | **median** | **Q1** | **Q3** | **Range** |
| --- | --- | --- | --- | --- |
| **C15:0** | 0.24 | 0.19 | 0.30 | 0.11-0.77 |
| C17:0 | 0.46 | 0.42 | 0.52 | 0.30-1.27 |
| C17:1n7 | **0.20** | 0.16 | 0.25 | 0.06-1.27 |
| **OCFAs** | **0.90** | 0.79 | 1.08 | 0.52-3.31 |

Notes: OCFAs were expressed as molar proportions (mol %) of total fatty acids. Abbreviation: OCFAs, odd-chain fatty acids; Q1, first quartile (25th percentile); Q3, third quartile (75th percentile).

Table S2. Stratified analysis of the association between plasma ln-transformed C15:0 levels and circulating WBC traits in psoriasis participants

| **Subgroups** | **WBC** | **Lymphocyte** | **Monocyte** | **Neutrophil** | **Basophil** | **Eosinophil** |
| --- | --- | --- | --- | --- | --- | --- |
| **sex** |  |  |  |  |  |  |
| Male | 0.85 (-0.12, 1.83) | 0.11 (-0.20, 0.41) | 0.04 (-0.05, 0.12) | 0.69 (-0.15, 1.53) | -0.01 (-0.04, 0.02) | -0.02 (-0.15, 0.11) |
| Female | **2.59 (0.87, 4.30)^**^** | 0.40 (-0.22, 1.02) | 0.04 (-0.10, 0.17) | 1.03 (-0.15, 2.22) | -0.01 (-0.06, 0.04) | **-1.10 (-2.05, -0.15)^*^** |
| **Age (year)** |  |  |  |  |  |  |
| < 40 | 0.88 (-0.51, 2.27) | **0.58 (0.12, 1.04)^*^** | 0.03 (-0.10, 0.16) | 0.20 (-0.88, 1.28) | 0.01 (-0.03, 0.04) | 0.07 (-0.31, 0.44) |
| ≥ 40 | **1.36 (0.28, 2.43)^*^** | 0.00 (-0.32, 0.32) | 0.03 (-0.05, 0.11) | **1.01 (0.12, 1.90)^*^** | -0.01 (-0.05, 0.02) | **-0.33 (-0.63, -0.02)^*^** |
| **BMI (kg/m^2^)** |  |  |  |  |  |  |
| < 24 | **1.60 (0.18, 3.01)^*^** | 0.19 (-0.24, 0.63) | 0.07 (-0.03, 0.17) | 0.79 (-0.34, 1.92) | 0.00 (-0.03, 0.02) | **-0.56 (-1.02, -0.10)^*^** |
| 24-28 | **1.28 (0.17, 2.38)^*^** | 0.16 (-0.26, 0.59) | -0.01 (-0.12, 0.10) | **1.02 (0.13, 1.90)^*^** | 0.00 (-0.02, 0.02) | 0.11 (-0.12, 0.34) |
| ≥ 28 | -0.96 (-4.22, 2.30) | -0.08 (-1.04, 0.88) | 0.01 (-0.27, 0.30) | -0.67 (-3.53, 2.18) | -0.10 (-0.28, 0.07) | -0.41 (-1.13, 0.31) |
| **Smoking history** | |  |  |  |  |  |
| No | 1.11 (-0.13, 2.35) | 0.35 (-0.09, 0.79) | 0.02 (-0.08, 0.12) | 0.74 (-0.23, 1.70) | 0.02 (-0.01, 0.04) | 0.01 (-0.22, 0.24) |
| Yes | **1.43 (0.22, 2.64)^*^** | -0.04 (-0.40, 0.32) | 0.05 (-0.05, 0.15) | 0.91 (-0.09, 1.91) | -0.03 (-0.07, 0.01) | **-0.48 (-0.85,-0.11)^**^** |
| **Alcohol consumption history** | |  |  |  |  |  |
| No | **1.40 (0.21, 2.59)^*^** | 0.15 (-0.21, 0.51) | 0.03 (-0.06, 0.13) | 0.80 (-0.15, 1.75) | -0.02 (-0.06, 0.02) | **-0.52(-0.89,-0.15)^**^** |
| Yes | 0.97 (-0.22, 2.16) | 0.18 (-0.26, 0.63) | 0.03 (-0.08, 0.13) | 0.64 (-0.39, 1.68) | 0.00 (-0.01, 0.02) | 0.10 (-0.03, 0.24) |
| **Psoriatic arthritis** | |  |  |  |  |  |
| No | **1.47 (0.60, 2.34)^**^** | 0.25 (-0.04, 0.54) | 0.06 (-0.02, 0.13) | **0.86 (0.16, 1.56)^*^** | -0.01 (-0.03, 0.02) | -0.26 (-0.53, 0.01) |
| **Hypertension** |  |  |  |  |  |  |
| No | 0.70 (-0.37, 1.78) | 0.23 (-0.13, 0.59) | 0.05 (-0.03, 0.14) | 0.44 (-0.44, 1.31) | 0.00 (-0.01, 0.02) | 0.00 (-0.16, 0.16) |
| **Type 2 diabetes** | |  |  |  |  |  |
| No | **1.00 (0.09, 1.92)^*^** | 0.13 (-0.16, 0.43) | 0.03 (-0.05, 0.10) | 0.59 (-0.15, 1.33) | 0.00 (-0.02, 0.01) | -0.24 (-0.50, 0.03) |

Notes: Data expressed as β (95% confidence interval). Significant results were presented in bold. The regression coefficients were adjusted for age, sex, BMI, education attainment, smoking history, alcohol consumption history, psoriasis duration, and psoriatic comorbidities including psoriatic arthritis, hypertension and type 2 diabetes. ^*^*P* < 0.05, ^**^*P* < 0.01. Abbreviation: BMI, body mass index; WBC, white blood cell.

Table S3. Stratified analysis of the association between plasma ln-transformed C17:0 levels and circulating WBC traits in psoriasis participants

| **Subgroups** | **WBC** | **Lymphocyte** | **Monocyte** | **Neutrophil** | **Basophil** | **Eosinophil** |
| --- | --- | --- | --- | --- | --- | --- |
| **Sex** |  |  |  |  |  |  |
| Male | 0.92 (-0.84, 2.68) | 0.30 (-0.24, 0.84) | 0.00 (-0.15, 0.15) | 0.54 (-0.98, 2.06) | -0.01 (-0.06, 0.04) | -0.03 (-0.27, 0.20) |
| Female | **5.06 (0.33, 9.79)^*^** | 0.62 (-1.02, 2.26) | 0.17 (-0.18, 0.52) | 1.24 (-1.95, 4.43) | -0.06 (-0.19, 0.07) | **-2.87 (-5.31, -0.43)^*^** |
| **Age (year)** |  |  |  |  |  |  |
| < 40 | -0.83 (-2.94, 1.29) | 0.44 (-0.29, 1.16) | -0.07 (-0.27, 0.13) | -1.14 (-2.75, 0.46) | -0.02 (-0.07, 0.03) | -0.13 (-0.70, 0.44) |
| ≥ 40 | **2.44 (0.13, 4.75)^*^** | 0.26 (-0.43, 0.95) | 0.06 (-0.12, 0.24) | 1.65 (-0.26, 3.56) | -0.02 (-0.09, 0.05) | -0.31 (-0.97, 0.35) |
| **BMI (kg/m^2^)** |  |  |  |  |  |  |
| < 24 | 2.04 (-1.60, 5.69) | 0.80 (-0.29, 1.89) | 0.06 (-0.20, 0.32) | 0.31 (-2.59, 3.20) | -0.01 (-0.06, 0.05) | **-**0.79 (-1.96,0.38) |
| 24-28 | 0.37 (-1.47, 2.21) | 0.17 (-0.53, 0.87) | -0.10 (-0.28, 0.08) | -0.01 (-1.47, 1.48) | -0.02 (-0.06, 0.01) | -0.03 (-0.41, 0.34) |
| ≥ 28 | 0.87 (-3.55, 5.28) | 0.04 (-1.25, 1.33) | 0.28 (-0.09, 0.65) | 1.00 (-2.85, 4.85) | 0.02 (-0.23, 0.26) | -0.06 (-1.05, 0.93) |
| **Smoking history** | |  |  |  |  |  |
| No | -0.17 (-2.54, 2.21) | 0.24 (-0.59, 1.08) | -0.01 (-0.20, 0.19) | -0.36 (-2.21, 1.48) | 0.00 (-0.05, 0.04) | -0.05 (-0.47, 0.37) |
| Yes | 2.16 (-0.24, 4.56) | 0.06 (-0.64, 0.77) | 0.00 (-0.20, 0.19) | 1.33 (-0.66, 3.32) | -0.04 (-0.12, 0.04) | **-0.55 (-1.29, 0.19)^**^** |
| **Alcohol consumption history** | |  |  |  |  |  |
| No | 1.32 (-1.04, 3.68) | 0.22 (-0.49, 0.92) | 0.03 (-0.16, 0.21) | 0.65 (-1.23, 2.54) | -0.03 (-0.10, 0.05) | -0.63(-1.36, 0.10) |
| Yes | 0.77 (-1.41, 2.95) | 0.31 (-0.50, 1.12) | -0.04 (-0.23, 0.15) | 0.16 (-1.73, 2.04) | 0.00 (-0.03, 0.02) | 0.14 (-0.11, 0.39) |
| **Psoriatic arthritis** | |  |  |  |  |  |
| No | 1.61(-0.09, 3.32) | 0.46 (-0.09, 1.01) | 0.06 (-0.08, 0.20) | 0.71 (-0.64, 2.07) | 0.00 (-0.06, 0.05) | -0.24 (-0.76, 0.28) |
| **Hypertension** |  |  |  |  |  |  |
| No | 0.33 (-1.59, 2.24) | 0.30 (-0.33, 0.94) | 0.04 (-0.11, 0.19) | 0.04 (-1.52, 1.60) | -0.01 (-0.04, 0.02) | -0.07 (-0.35, 0.21) |
| **Type 2 diabetes** | |  |  |  |  |  |
| No | 0.65 (-1.10, 2.40) | 0.37 (-0.19, 0.92) | 0.01 (-0.13, 0.16) | 0.09 (-1.31, 1.50) | -0.01 (-0.04, 0.01) | -0.25 (-0.75, 0.25) |

Notes: Data expressed as β (95% confidence interval). Significant results were presented in bold. The regression coefficients were adjusted for age, sex, BMI, education attainment, smoking history, alcohol consumption history, psoriasis duration, and psoriatic comorbidities including psoriatic arthritis, hypertension and type 2 diabetes. ^*^*P* < 0.05, ^**^*P* < 0.01. Abbreviation: BMI, body mass index; WBC, white blood cell.

Table S4. Stratified analysis of the association between plasma ln-transformed C17:1n7 levels and circulating WBC traits in psoriasis participants

| **Subgroups** | **WBC** | **Lymphocyte** | **Monocyte** | **Neutrophil** | **Basophil** | **Eosinophil** |
| --- | --- | --- | --- | --- | --- | --- |
| **Sex** |  |  |  |  |  |  |
| Male | 0.46 (-0.41, 1.33) | 0.23 (-0.03, 0.50) | **0.08 (0.00, 0.15)^*^** | 0.15 (-0.60, 0.90) | 0.01 (-0.02, 0.03) | 0.02 (-0.10, 0.13) |
| Female | **2.06 (0.04, 4.08)^*^** | 0.62 (-0.06, 1.30) | 0.06 (-0.09, 0.20) | 0.75 (-0.59, 2.10) | 0.01 (-0.04, 0.07) | -0.48 (-1.60, 0.65) |
| **Age (year)** |  |  |  |  |  |  |
| < 40 | 0.33 (-0.78, 1.44) | 0.37 (-0.01, 0.74) | 0.01 (-0.09, 0.12) | -0.07 (-0.93, 0.78) | -0.01 (-0.03, 0.02) | -0.02 (-0.31, 0.28) |
| ≥ 40 | 0.85 (-0.23, 1.93) | 0.21 (-0.11, 0.53) | **0.08 (0.00, 0.16)^*^** | 0.47 (-0.42, 1.37) | 0.02 (-0.01, 0.05) | -0.02 (-0.33, 0.29) |
| **BMI (kg/m^2^)** |  |  |  |  |  |  |
| < 24 | 1.00 (-0.53, 2.54) | 0.42 (-0.03, 0.87) | 0.05 (-0.06, 0.16) | 0.33 (-0.88, 1.55) | 0.01 (-0.01, 0.03) | -0.11 (-0.62, 0.39) |
| 24-28 | **1.19 (0.24, 2.13)^*^** | 0.31 (-0.06, 0.67) | 0.05 (-0.04, 0.15) | **0.79 (0.03, 1.56)^*^** | -0.00 (-0.02, 0.02) | -0.03 (-0.23, 0.16) |
| ≥ 28 | -1.59 (-3.62, 0.45) | 0.09 (-0.53, 0.70) | 0.12 (-0.06, 0.30) | -1.68 (-3.44, 0.07) | 0.02 (-0.10, 0.13) | 0.00 (-0.47, 0.48) |
| **Smoking history** | |  |  |  |  |  |
| No | 0.81 (-0.21, 1.83) | **0.49 (0.14, 0.85)^**^** | 0.03 (-0.06, 0.11) | 0.28 (-0.52, 1.09) | 0.01 (-0.01, 0.02) | -0.02 (-0.20, 0.17) |
| Yes | 0.36 (-0.96, 1.68) | -0.04 (-0.43, 0.35) | 0.11 (0.00, 0.21) | 0.04 (-1.06, 1.13) | 0.01 (-0.03, 0.06) | -0.13 (-0.54, 0.27) |
| **Alcohol consumption history** | |  |  |  |  |  |
| No | **1.12 (0.04, 2.20)^*^** | 0.45 (0.14, 0.77) | 0.08 (0.00, 0.17) | 0.47 (-0.40, 1.34) | 0.01 (-0.03, 0.04) | -0.10 (-0.44, 0.25) |
| Yes | 0.00 (-1.14, 1.15) | 0.09 (-0.33, 0.51) | 0.05 (-0.05, 0.15) | -0.18 (-1.16, 0.81) | 0.01 (-0.01, 0.02) | 0.05 (-0.08, 0.18) |
| **Psoriatic arthritis** | |  |  |  |  |  |
| No | **0.85 (0.04, 1.67)^*^** | **0.42 (0.16, 0.67)^**^** | **0.07 (0.00, 0.14)^*^** | 0.31 (-0.34, 0.96) | 0.01 (-0.01, 0.04) | -0.02 (-0.27, 0.23) |
| **Hypertension** |  |  |  |  |  |  |
| No | 0.42 (-0.56, 1.39) | **0.49 (0.17, 0.80)^**^** | 0.05 (-0.03, 0.13) | -0.13 (-0.93, 0.66) | 0.00 (-0.01, 0.02) | -0.01 (-0.16, 0.13) |
| **Type 2 diabetes** | |  |  |  |  |  |
| No | 0.54 (-0.30, 1.38) | **0.33 (0.06, 0.59)^*^** | 0.07 (0.00, 0.13) | 0.12 (-0.56, 0.80) | 0.00 (-0.01, 0.02) | -0.02 (-0.26, 0.22) |

Notes: Data expressed as β (95% confidence interval). Significant results were presented in bold. The regression coefficients were adjusted for age, sex, BMI, education attainment, smoking history, alcohol consumption history, psoriasis duration, and psoriatic comorbidities including psoriatic arthritis, hypertension and type 2 diabetes. ^*^*P* < 0.05, ^**^*P* < 0.01. Abbreviation: BMI, body mass index; WBC, white blood cell.

Table S5. Stratified analysis of the association between plasma ln-transformed total odd-chain fatty acids levels and circulating WBC traits in psoriasis participants

| **Subgroups** | **WBC** | **Lymphocyte** | **Monocyte** | **Neutrophil** | **Basophil** | **Eosinophil** |
| --- | --- | --- | --- | --- | --- | --- |
| **Sex** |  |  |  |  |  |  |
| Male | 1.00 (-0.36, 2.36) | 0.27 (-0.14, 0.69) | 0.06 (-0.06, 0.17) | 0.64 (-0.54, 1.81) | 0.00 (-0.04, 0.04) | -0.01 (-0.19, -0.17) |
| Female | **4.15 (1.21, 7.09)^**^** | 0.79 (-0.24, 1.82) | 0.10 (-0.12, 0.32) | 1.48 (-0.53, 3.50) | -0.01 (-0.10, 0.07) | **-1.66 (-3.28, -0.05)^*^** |
| **Age (year)** |  |  |  |  |  |  |
| < 40 | 0.18 (-1.52, 1.87) | 0.55 (-0.01, 1.12) | -0.01 (-0.17, 0.14) | -0.38 (-1.68, 0.92) | -0.01 (-0.05, 0.03) | -0.04 (-0.49, 0.41) |
| ≥ 40 | **2.11 (0.42, 3.80)^*^** | 0.19 (-0.31, 0.70) | 0.09 (-0.04, 0.22) | **1.47 (0.07, 2.87)^*^** | 0.00 (-0.05, 0.05) | -0.29 (-0.78, 0.20) |
| **BMI (kg/m^2^)** |  |  |  |  |  |  |
| < 24 | 2.22 (-0.20, 4.64) | 0.54 (-0.19, 1.27) | 0.10 (-0.08, 0.27) | 0.89 (-1.05, 2.82) | 0.00 (-0.03, 0.04) | -0.64 (-1.43, 0.15) |
| 24-28 | 1.34 (-0.11, 2.79) | 0.31 (-0.24, 0.87) | -0.01 (-0.16, 0.14) | 0.88 (-0.30, 2.05) | -0.01 (-0.04, 0.02) | 0.02 (-0.28, 0.32) |
| ≥ 28 | -0.96 (-4.68, 2.75) | 0.06 (-1.03, 1.14) | 0.18 (-0.14, 0.50) | -0.87 (-4.11, 2.38) | -0.02 (-0.22, 0.19) | -0.16 (-0.99, 0.67) |
| **Smoking history** | |  |  |  |  |  |
| No | 1.00 (-0.70, 2.69) | 0.54 (-0.06, 1.13) | 0.02 (-0.12, 0.16) | 0.44 (-0.89, 1.76) | 0.01 (-0.02, 0.04) | -0.02 (-0.32, 0.29) |
| Yes | 1.89 (-0.03, 3.81) | -0.02 (-0.58, 0.55) | 0.08 (-0.07, 0.24) | 1.14 (-0.45, 2.74) | -0.03 (-0.09, 0.04) | -0.54 (-1.13, 0.05) |
| **Alcohol consumption history** | |  |  |  |  |  |
| No | **1.89 (0.12, 3.66)^*^** | 0.41 (-0.12, 0.95) | 0.08 (-0.06, 0.22) | 0.98 (-0.43, 2.40) | -0.01 (-0.07, 0.04) | -0.53 (-1.08, 0.03) |
| Yes | 0.78 (-0.92, 2.48) | 0.26 (-0.37, 0.89) | 0.02 (-0.13, 0.17) | 0.32 (-1.15, 1.79) | 0.00 (-0.02, 0.02) | 0.12 (-0.07, 0.31) |
| **Psoriatic arthritis** | |  |  |  |  |  |
| No | **1.78 (0.50, 3.05)^**^** | **0.50 (0.09, 0.91)^*^** | 0.09 (-0.02, 0.19) | 0.89 (-0.12, 1.91) | 0.00 (-0.04, 0.04) | -0.21 (-0.60, 0.18) |
| **Hypertension** |  |  |  |  |  |  |
| No | 0.75 (-0.77, 2.27) | 0.48 (-0.02, 0.98) | 0.07 (-0.05, 0.19) | 0.22 (-1.02, 1.46) | 0.00 (-0.03, 0.02) | -0.04 (-0.26, 0.19) |
| **Type 2 diabetes** | |  |  |  |  |  |
| No | 1.08 (-0.25, 2.40) | 0.37 (-0.05, 0.79) | 0.06 (-0.05, 0.16) | 0.47 (-0.61, 1.54) | 0.00 (-0.03, 0.02) | -0.21 (-0.59, 0.17) |

Notes: Data expressed as β (95% confidence interval). Significant results were presented in bold. The regression coefficients were adjusted for age, sex, BMI, education attainment, smoking history, alcohol consumption history, psoriasis duration, and psoriatic comorbidities including psoriatic arthritis, hypertension and type 2 diabetes. ^*^*P* < 0.05, ^**^*P* < 0.01. Abbreviation: BMI, body mass index; WBC, white blood cell.

Table S6. Stratified analysis of the association between plasma ln-transformed C15:0 levels and psoriasis severity in psoriasis participants

| **Subgroups** | **PASI** | **BSA** | **DLQI** | **HADS-A** | **HADS-D** |
| --- | --- | --- | --- | --- | --- |
| **Sex** |  |  |  |  |  |
| Male | -1.21 (-4.43, 2.01) | -0.76 (-8.08, 6.55) | 0.58 (-2.36, 3.53) | -0.07 (-2.14,2.00) | -1.10 (-3.10, 0.90) |
| Female | -2.82 (-6.72, 1.08) | -2.67 (-10.60, 5.26) | 0.82 (-4.45, 6.09) | -1.44 (-5.35, 2.46) | -1.90 (-5.37, 1.57) |
| **Age (year)** |  |  |  |  |  |
| < 40 | -0.83 (-5.04, 3.39) | -1.68 (-10.25, 6.90) | -1.07 (-6.37, 4.23) | -2.27 (-5.54, 1.01) | -1.84 (-4.88, 1.20) |
| ≥ 40 | -1.75 (-5.11, 1.61) | -1.00 (-8.57, 6.58) | 1.45 (-1.64, 4.53) | 0.46 (-1.82, 2.74) | -0.95 (-3.12, 1.22) |
| **BMI (kg/m^2^)** |  |  |  |  |  |
| < 24 | -1.53 (-5.25, 2.18) | 1.00 (-7.08, 9.09) | 2.39 (-1.07, 5.85) | 0.42 (-2.19, 3.02) | -0.05 (-2.20, 2.10) |
| 24-28 | -1.68 (-6.23, 2.86) | -2.62 (-13.14, 7.90) | -1.17 (-5.62, 3.32) | -1.80 (-4.88, 1.28) | **-3.37(-6.61, -0.13)^*^** |
| ≥ 28 | -3.79(-12.47, 4.88) | -7.28(-26.25, 11.68) | 2.24 (-8.48, 12.96) | 4.89 (-2.22, 11.99) | 2.76 (-4.26, 9.78) |
| **Smoking history** | |  |  |  |  |
| No | -0.62 (-4.04, 2.79) | 2.05 (-6.68, 10.77) | 0.92 (-3.01, 4.86) | -0.26 (-2.95, 2.42) | -1.82 (-4.29, 0.64) |
| Yes | -2.07 (-6.03, 1.88) | -2.82 (-11.39, 5.76) | 0.78 (-2.86, 4.41) | -0.46 (-3.05, 2.13) | -1.08 (-3.59, 1.43) |
| **Alcohol consumption history** | |  |  |  |  |
| No | -2.76 (-6.13, 0.62) | -4.01 (-11.66, 3.64) | 1.15 (-2.22, 4.51) | -0.59 (-2.84, 1.65) | -1.65 (-3.72, 0.41) |
| Yes | 0.54 (-3.93, 5.01) | 4.62 (-5.55, 14.80) | 0.50 (-3.94, 4.94) | 0.46 (-3.01, 3.93) | 0.14 (-3.15, 3.43) |
| **Psoriatic arthritis** | |  |  |  |  |
| No | -1.92 (-4.73, 0.89) | -1.68 (-8.05, 4.70) | 1.86 (-0.81, 4.54) | -0.11 (-2.06, 1.83) | -0.68 (-2.47, 1.12) |
| **Hypertension** |  |  |  |  |  |
| No | -1.55 (-4.78, 1.67) | -2.28 (-9.42, 4.86) | 1.24 (-1.97, 4.44) | -0.47 (-2.46, 1.51) | -0.70 (-2.59, 1.19) |
| **Type 2 diabetes** | |  |  |  |  |
| No | -1.31 (-4.09, 1.47) | -1.19 (-7.59, 5.22) | 1.08 (-1.66, 3.82) | -0.54 (-2.46, 1.38) | -1.15 (-2.99, 0.70) |

Notes: Data expressed as β (95% confidence interval). Significant results were presented in bold. The regression coefficients were adjusted for age, sex, body mass index, education attainment, smoking history, alcohol consumption history, psoriasis duration, and psoriatic comorbidities including psoriatic arthritis, hypertension and type 2 diabetes. ^*^*P* < 0.05. Abbreviation: BMI, body mass index; BSA, body surface area; DLQI, dermatology life quality index; HADS-A, hospital anxiety and depression scale for anxiety; HADS-D, hospital anxiety and depression scale for depression; PASI, psoriasis area and severity index.

Table S7. Stratified analysis of the association between plasma ln-transformed C17:0 levels and psoriasis severity in psoriasis participants

| **Subgroups** | **PASI** | **BSA** | **DLQI** | **HADS-A** | **HADS-D** |
| --- | --- | --- | --- | --- | --- |
| **Sex** |  |  |  |  |  |
| Male | -1.17 (-6.98, 4.63) | -5.07 (-18.44, 8.30) | -2.15 (-7.44, 3.15) | -1.56 (-5.24, 2.12) | -2.35 (-5.92, 1.22) |
| Female | **-10.22 (-20.27, -0.18)^*^** | -18.28 (-38.44, 1.89) | 4.44 (-9.17, 18.05) | 1.03 (-9.56, 11.62) | 1.08 (-8.39, 10.54) |
| **Age (year)** |  |  |  |  |  |
| < 40 | -2.73 (-9.05, 3.60) | -6.07 (-18.94, 6.80) | -6.90 (-14.67, 0.88) | **-5.33 (-10.15, -0.51)^*^** | -3.88 (-8.39, 0.63) |
| ≥ 40 | -2.59 (-9.80, 4.62) | -7.16 (-23.67, 9.35) | 0.57 (-6.05, 7.19) | 0.88 (-4.15, 5.92) | -1.95 (-6.73, 2.84) |
| **BMI (kg/m^2^)** |  |  |  |  |  |
| < 24 | -1.61 (-11.03, 7.81) | 0.83 (-19.61, 21.26) | 3.09 (-5.80, 11.98) | -0.96 (-7.64, 5.72) | -0.18 (-5.71,5.34) |
| 24-28 | -3.39 (-10.77, 4.00) | -10.82 (-27.82, 6.17) | -5.10 (-12.03, 1.84) | -2.16 (-6.95, 2.63) | -2.75 (-7.87, 2.36) |
| ≥ 28 | -4.55 (-16.36, 7.27) | -15.88(-43.82,12.05) | -2.42(-16.67, 11.83 ) | 2.53( -7.38, 12.44 ) | -3.32(-12.83, 6.20 ) |
| **Smoking history** | |  |  |  |  |
| No | -0.92 (-7.35, 5.51) | -3.51 (-20.52, 13.49) | -0.76 (-8.19, 6.67) | 0.19 (-4.78, 5.16) | -1.92 (-6.52, 2.69) |
| Yes | -4.17 (-11.97, 3.64) | -9.80 (-26.66, 7.05) | -2.89 (-9.99, 4.21) | -3.36 (-8.46, 1.74) | -3.26 (-8.23, 1.71) |
| **Alcohol consumption history** | |  |  |  |  |
| No | -4.25 (-10.90, 2.39) | -11.84 (-27.22, 3.55) | -2.36 (-9.08, 4.35) | -2.68 (-7.12, 1.75) | -3.63 (-7.72, 0.46) |
| Yes | -1.30 (-9.40, 6.81) | -1.06 (-19.61, 17.50) | -3.23 (-11.06, 4.60) | -0.48 (-6.52, 5.57) | -0.36 (-6.09, 5.38) |
| **Psoriatic arthritis** | |  |  |  |  |
| No | -3.47 (-8.89, 1.94) | -8.92 (-21.15, 3.30) | -1.22 (-6.37, 3.92) | -2.36 (-6.09, 1.37) | -2.36 (-5.81, 1.09) |
| **Hypertension** |  |  |  |  |  |
| No | -2.96 (-8.68, 2.75) | -8.75 (-21.66, 4.17) | -1.99 (-7.76, 3.78) | -2.27 (-5.75, 1.21) | -1.99 (-5.31, 1.34) |
| **Type 2 diabetes** | |  |  |  |  |
| No | -2.15 (-7.41, 3.10) | -7.83 (-20.07, 4.42) | -2.80 (-8.00, 2.40) | -2.16 (-5.76, 1.43) | -2.16 (-5.62, 1.31) |

Notes: Data expressed as β (95% confidence interval). Significant results were presented in bold. The regression coefficients were adjusted for age, sex, BMI, education attainment, smoking history, alcohol consumption history, psoriasis duration, and psoriatic comorbidities including psoriatic arthritis, hypertension and type 2 diabetes. ^*^*P* < 0.05. Abbreviation: BMI, body mass index; BSA, body surface area; DLQI, dermatology life quality index; HADS-A, hospital anxiety and depression scale for anxiety; HADS-D, hospital anxiety and depression scale for depression; PASI, psoriasis area and severity index.

Table S8. Stratified analysis of the association between plasma ln-transformed C17:1n7 levels and psoriasis severity in psoriasis participants

| **Subgroups** | **PASI** | **BSA** | **DLQI** | **HADS-A** | **HADS-D** |
| --- | --- | --- | --- | --- | --- |
| **Sex** |  |  |  |  |  |
| Male | -0.84 (-3.70, 2.01) | -0.98 (-7.52, 5.55) | 1.31 (-1.28, 3.90) | -0.06 (-1.85, 1.73) | -0.68 (-2.41, 1.05) |
| Female | -1.98 (-6.40, 2.44) | -2.64 (-11.47, 6.18) | 1.29 (-4.90, 7.48) | -2.10 (-7.14, 2.94) | -1.73 (-6.25, 2.80) |
| **Age (year)** |  |  |  |  |  |
| < 40 | -1.23 (-4.55, 2.10) | -1.63 (-8.41, 5.16) | -1.19 (-5.38, 3.01) | -2.03 (-4.61, 0.54) | -1.86 (-4.24, 0.53) |
| ≥ 40 | -1.18 (-4.53, 2.17) | -1.50 (-9.12, 6.13) | 2.82 (-0.27, 5.90) | 0.80 (-1.53, 3.13) | -0.05 (-2.27, 2.18) |
| **BMI (kg/m^2^)** |  |  |  |  |  |
| < 24 | -0.89 (-4.85, 3.06) | -0.81 (-9.41, 7.79) | 1.98 (-1.92, 5.88) | -0.67 (-3.57, 2.24) | 0.13 (-2.27, 2.54) |
| 24-28 | -2.05 (-5.95, 1.84) | -3.50 (-12.53, 5.53) | 0.19 (-3.56, 3.95) | -0.45 (-3.04, 2.15) | -1.36 (-4.13, 1.40) |
| ≥ 28 | 1.19 (-4.39, 6.77) | 2.67 (-9.99, 15.32) | 4.70 (-1.98, 11.38) | 2.39 (-2.32, 7.11) | 0.73 (-3.90, 5.35) |
| **Smoking history** | |  |  |  |  |
| No | 0.69 (-2.11, 3.49) | 1.54 (-5.74, 8.81) | 0.78 (-2.57, 4.14) | -0.45 (-2.75, 1.86) | -0.79 (-2.93, 1.35) |
| Yes | -4.03 (-8.26, 0.20) | -6.27 (-15.45, 2.90) | 2.02 (-1.88, 5.91) | -1.20 (-3.92, 1.51) | -1.53 (-4.17, 1.10) |
| **Alcohol consumption history** | |  |  |  |  |
| No | -2.67 (-5.73, 0.39) | -5.07 (-12.09, 1.94) | 1.17 (-1.94, 4.29) | -0.68 (-2.80, 1.44) | -0.59 (-2.56, 1.37) |
| Yes | 0.57 (-3.65, 4.80) | 3.63 (-6.01, 13.26) | 1.54 (-2.59, 5.66) | 0.23 (-2.88, 3.33) | -0.30 (-3.24, 2.65) |
| **Psoriatic arthritis** | |  |  |  |  |
| No | -1.63 (-4.22, 0.95) | -2.57 (-8.43, 3.28) | 1.29 (-1.19, 3.78) | -0.48 (-2.28, 1.32) | -0.56 (-2.22, 1.11) |
| **Hypertension** |  |  |  |  |  |
| No | -0.61 (-3.52, 2.30) | -1.85 (-8.37, 4.67) | 1.07 (-1.87, 4.02) | -0.75 (-2.54, 1.04) | -0.43 (-2.14, 1.27) |
| **Type 2 diabetes** | |  |  |  |  |
| No | -0.97 (-3.51, 1.56) | -2.53 (-8.40, 3.34) | 1.07 (-1.46, 3.59) | -0.81 (-2.56, 0.95) | -0.74 (-2.43, 0.96) |

Notes: Data expressed as β (95% confidence interval). Significant results were presented in bold. The regression coefficients were adjusted for age, sex, BMI, education attainment, smoking history, alcohol consumption history, psoriasis duration, and psoriatic comorbidities including psoriatic arthritis, hypertension and type 2 diabetes. Abbreviation: BMI, body mass index; BSA, body surface area; DLQI, dermatology life quality index; HADS-A, hospital anxiety and depression scale for anxiety; HADS-D, hospital anxiety and depression scale for depression; PASI, psoriasis area and severity index.

Table S9. Stratified analysis of the association between plasma ln-transformed total odd-chain fatty acids levels and psoriasis severity in psoriasis participants

| **Subgroups** | **PASI** | **BSA** | **DLQI** | **HADS-A** | **HADS-D** |
| --- | --- | --- | --- | --- | --- |
| **Sex** |  |  |  |  |  |
| Male | -1.59 (-6.07, 2.90) | -2.77 (-13.05, 7.50) | 0.25 (-3.84, 4.34) | -0.62 (-3.45, 2.20) | -1.66 (-4.40, 1.07) |
| Female | -5.36 (-11.92, 1.19) | -7.72 (-20.95, 5.51) | 2.39 (-6.64, 11.42) | -1.86 (-8.91, 5.18) | -2.10 (-8.41, 4.20) |
| **Age (year)** |  |  |  |  |  |
| < 40 | -1.90 (-6.96, 3.15) | -3.66 (-13.94, 6.61) | -3.37 (-9.68, 2.95) | -3.70 (-7.58, 0.18) | -3.00 (-6.61, 0.61) |
| ≥ 40 | -2.65 (-7.95, 2.65) | -3.71 (-15.79, 8.36) | 2.63 (-2.25, 7.52) | 0.97 (-2.69, 4.63) | -1.19 (-4.68, 2.29) |
| **BMI (kg/m^2^)** |  |  |  |  |  |
| < 24 | -2.09 (-8.39, 4.22) | 0.31 (-13.39, 14.01) | 3.47 (-2.50, 9.44) | -0.33 (-4.77,4.11) | -0.08 (-3.75, 3.59) |
| 24-28 | -3.12 (-9.04, 2.79) | -7.04 (-20.71, 6.63) | -2.19 (-7.87, 3.50) | -1.83 (-5.74, 2.08) | -3.12 (-7.26, 1.03) |
| ≥ 28 | -2.32 (-12.31, 7.66) | -6.79(-29.87, 16.30) | 3.35 (-8.73, 15.43) | 4.51 (-3.68, 12.71) | 0.47 (-7.59, 8.52) |
| **Smoking history** | |  |  |  |  |
| No | -0.30 (-4.93, 4.33) | 0.58 (-11.46, 12.63) | 0.54 (-4.88, 5.97) | -0.44 (-4.12, 3.24) | -1.95 (-5.34, 1.45) |
| Yes | -4.79 (-11.04, 1.45) | -8.37 (-21.89, 5.16) | 0.56 (-5.18, 6.31) | -1.96 (-6.02, 2.09) | -2.48 (-6.42, 1.45) |
| **Alcohol consumption history** | |  |  |  |  |
| No | -4.58 (-9.58, 0.42) | -9.25 (-20.73, 2.24) | 0.78 (-4.29, 5.86) | -1.47 (-4.84, 1.90) | -2.32 (-5.43, 0.79) |
| Yes | 0.08 (-6.23, 6.39) | 3.76(-10.63, 18.16) | -0.29 (-6.47, 5.89) | -0.02 (-4.73, 4.70) | -0.32 (-4.79, 4.15) |
| **Psoriatic arthritis** | |  |  |  |  |
| No | -3.10 (-7.17, 0.97) | -5.23 (-14.45, 4.00) | 1.26 (-2.63, 5.16) | -1.10 (-3.92, 1.71) | -1.44 (-4.04, 1.16) |
| **Hypertension** |  |  |  |  |  |
| No | -2.39 (-6.93, 2.14) | -5.70 (-15.88, 4.47) | 0.59 (-3.98, 5.15) | -1.45 (-4.23, 1.33) | -1.23 (-3.88, 1.42) |
| **Type 2 diabetes** | |  |  |  |  |
| No | -1.99 (-6.00, 2.01) | -4.68 (-13.98, 4.62) | 0.26 (-3.71, 4.24) | -1.43 (-4.18, 1.32) | -1.70 (-4.35, 0.95) |

Notes: Data expressed as β (95% confidence interval). Significant results were presented in bold. The regression coefficients were adjusted for age, sex, BMI, education attainment, smoking history, alcohol consumption history, psoriasis duration, and psoriatic comorbidities including psoriatic arthritis, hypertension and type 2 diabetes. Abbreviation: BMI, body mass index; BSA, body surface area; DLQI, dermatology life quality index; HADS-A, hospital anxiety and depression scale for anxiety; HADS-D, hospital anxiety and depression scale for depression; PASI, psoriasis area and severity index.

##
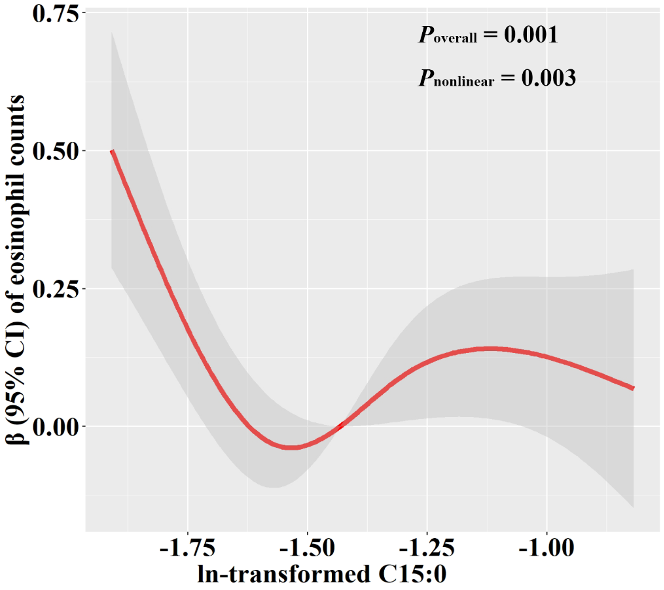
Supplementary Figures

**Figure S1.** Predicted spline curves for the associations between plasma ln-transformed C15:0 levels and eosinophil count among all participants using RCS regression models. Abbreviation: ln, natural log-transformed; RCS, restricted cubic spline.
